# Paraneoplastic neuromyelitis optica spectrum disorder associated with ovarian dysgerminoma: a case report and literature review

**DOI:** 10.3389/fimmu.2024.1424243

**Published:** 2024-06-14

**Authors:** Pan Liu, Shuangying Wang, Chunhua Zhang, Yanfang Li

**Affiliations:** ^1^ Department of Neurology, The Central Hospital of Shaoyang, Shaoyang, China; ^2^ Department of Clinical Pharmacy, The Central Hospital of Shaoyang, Shaoyang, China

**Keywords:** paraneoplastic NMOSD, AQP4, ovarian dysgerminoma, immunosuppression, IVMP

## Abstract

Neuromyelitis optica spectrum disorder (NMOSD) is a clinical syndrome characterized by attacks of acute optic neuritis and transverse myelitis. We report a case with paraneoplastic NMOSD that improved after immunosuppressive therapy, surgical resection, and chemotherapy. A 48-year-old woman initially presented with gradual binocular visual loss over the course of one week. The patient was evaluated using magnetic resonance imaging (MRI), computed tomography (CT), visual evoked potential (VEP), pathological biopsy, immunohistochemistry, and autoimmune antibody testing. The brain MRI findings were normal. The VEP revealed prolonged P100 latencies in the right eye and an absence of significant waves in the left eye. Positive serum AQP4-IgG antibodies were found. The patient was diagnosed as NMOSD. Then the patient responded well to treatment with methylprednisolone. An ovarian tumor was found in the patient using abdominal MRI and CT. The tumor was surgically resected, and a pathological biopsy revealed that it was ovarian dysgerminoma. The patient received four rounds of chemotherapy after surgery. One month after the final chemotherapy treatment, a positron emission tomography (PET) scan revealed no tumor. The vision of the patient gradually recovered and serum AQP4 was negative. Furthermore, we summarized the characteristics of patients diagnosed with paraneoplastic NMOSD associated with ovarian neoplasms in previous studies. This is a characteristic case of overlapping NMOSD and ovarian dysgerminoma, demonstrating the importance of tumor therapy in cases of paraneoplastic NMOSD.

## 1 Introduction

Neuromyelitis optica spectrum disorder (NMOSD) is a relapsing inflammatory nervous system disease characterized by simultaneous or consecutive attacks of acute optic neuritis (ON) and transverse myelitis (TM) (1). IgG autoantibodies to aquaporin 4 (AQP4-IgG), the most abundant water channel protein in the central nervous system (CNS) (2), are currently the serological and pathophysiological marker of NMOSD (3). Ovarian dysgerminoma is a rare malignant tumor that originates from ovarian primordial germ cells, with an incidence of 0.109 per 100,000 women-years (4). Some studies have found an association between NMOSD and cancer, such as lung cancer, breast cancer, ovarian teratomas, etc. (5). To our knowledge, paraneoplastic NMOSD complicated with ovarian dysgerminoma is rare. Herein, we report a case with NMOSD overlapping ovarian dysgerminoma with an excellent prognosis.

## 2 Case report

A 48-year-old Chinese woman initially visited the Department of Ophthalmology at the Central Hospital of Shaoyang on February 15, 2022, due to a one-week history of visual impairment. She had a history of uterine fibroids and had a total hysterectomy 11 years ago. Upon physical examination, it was noted that she had no light perception in both eyes, and bilateral pupillary light reflexes were absent. Other ophthalmic tests conducted by ophthalmologists showed normal results. Consequently, the visual deterioration of the patient was considered as optic nerve or visual cortex damage.

**Figure 1:**
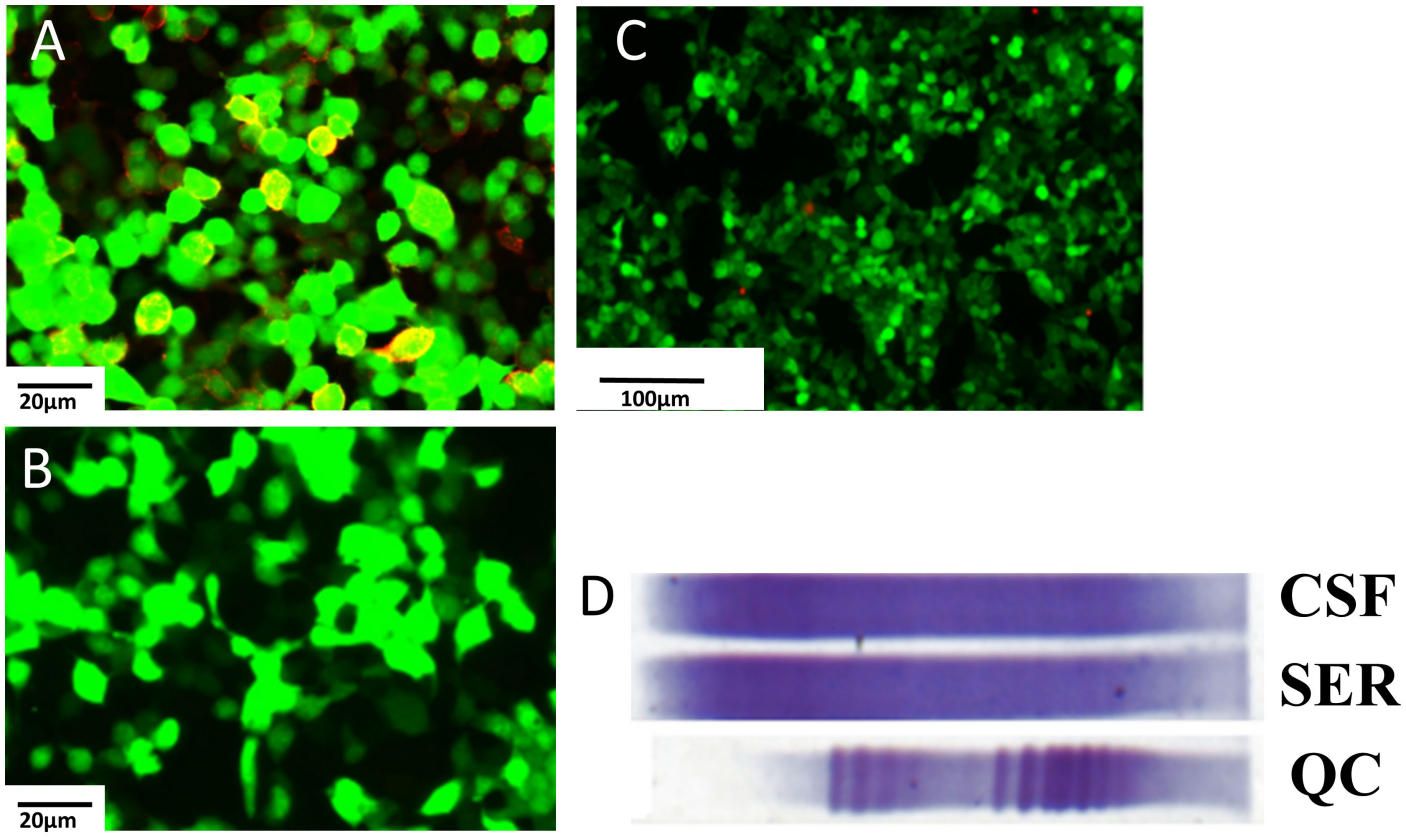
Demyelinating antibody of central nervous system. **(A)** AQP4 antibodies (1:10) are detected in the serum in the first test (scale bar 20μm). **(B)** AQP4 antibodies are found negative in the CSF (scale bar 20μm). **(C)** AQP4 antibodies are found negative in the serum in the second detect (scale bar 20μm). **(D)** OCBs are found in both serum and CSF, and the locations are identical. AQP4, aquaporin 4; CSF, cerebrospinal fluid; OCBs, oligoclonal bands.

Then the patient was referred to the Department of Neurology in our hospital for neurologic evaluation. A systemic neurological examination was conducted, revealing that muscle strength, muscle tension, tendon reflexes, sensory nervous system, cerebellar functions, Babinski signs, and meningeal irritation signs were all within normal limits. She underwent visual evoked potential (VEP) test, which showed prolonged P100 latencies in the right eye and no detectable P100 waves in the left eye. Magnetic resonance imaging (MRI) of eyes, optic nerves, orbits, brain, and spinal cord showed normal findings. A lumbar puncture was performed, which revealed normal intracranial pressure, white cell counts, glucose, and chloride levels in the cerebrospinal fluid (CSF). Then we completed the investigation with serum and CSF demyelinating antibodies (AQP4, MOG, GFAP, and MBP) screening by the cell-based assay (CBA) method (Kingmed Diagnostics Co., Ltd., which is the largest College of American Pathologists-certified laboratory in China) (6). The AQP4-IgG (antibody titer: 1:10, assessed by CBA method) was detected positive in the serum (**Figure 1A**), while no abnormalities were found in the CSF AQP4-IgG (**Figure 1B**). Additionally, we searched for IgG-oligoclonal bands (OCBs) in the CSF and serum using isoelectric focusing electrophoresis analysis. The patient’s serum and CSF contained identical IgG-OCBs. (**Figure 1D**). Therefore, according to IPND Criteria 2015, the patient was diagnosed as NMOSD by the clinical feature and the positive antibody.

The patient then underwent a course of treatment of methylprednisolone. The patient was on IVMP for 5 days (1,000 mg/day) (the dosage was decreased every 3 days), followed by oral prednisone (60 mg/day) and mycophenolate mofetil (1 g/day). The vision of patient gradually recovered after immunosuppressive therapy. She was continued on immunosuppressive treatment with prednisone (60 mg/day) (the dosage was decreased to 10mg/day) and mycophenolate mofetil (1 g/day) after discharge.

**Figure 2:**
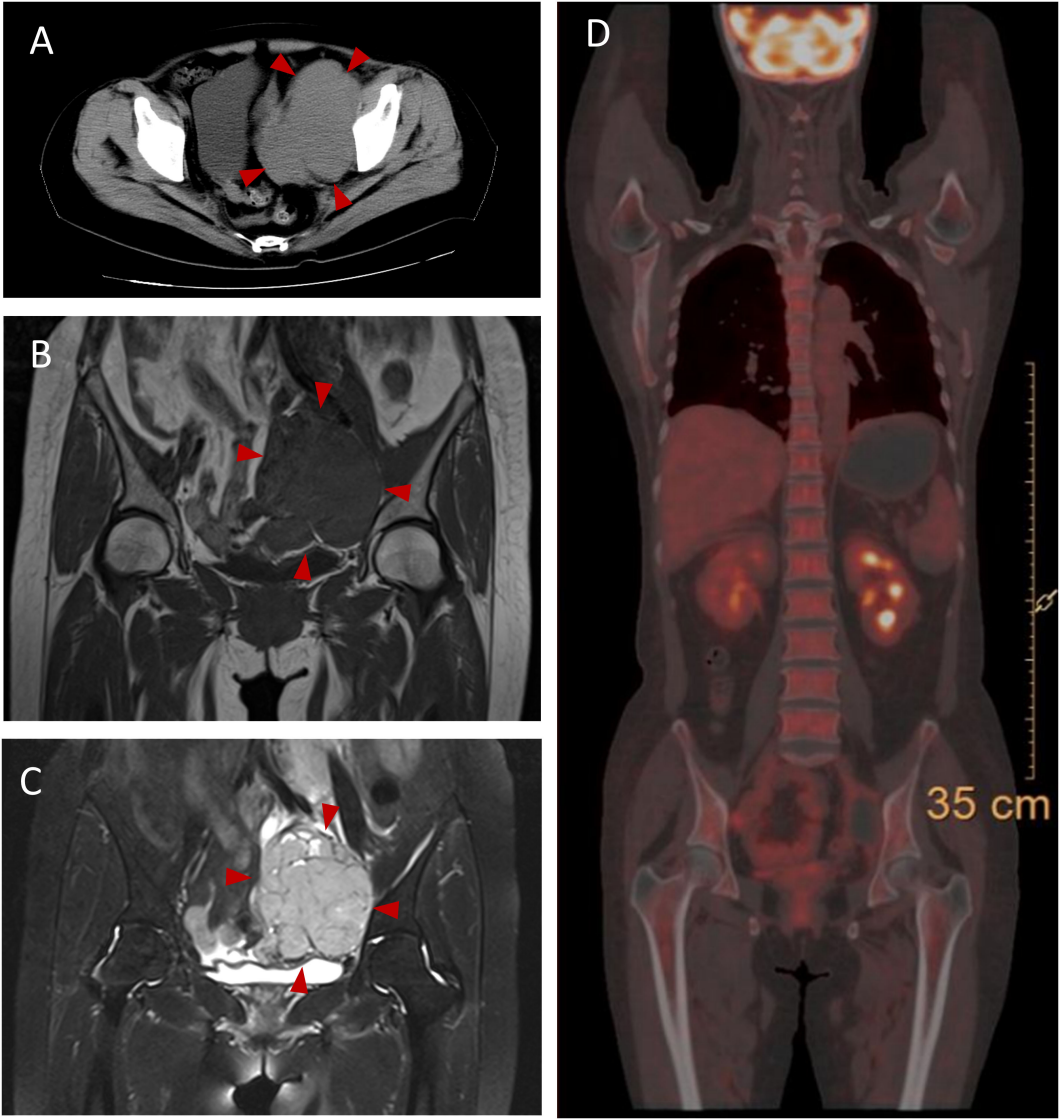
Abdominal CT and MRI and PET-CT findings in this case. **(A)** Abdominal CT shows an equal-denstiy mass pointed by the red arrowheads in the left lower abdomen. **(B)** T1-weighted image shows a hypointense mass pointed by the red arrowheads in the left lower abdomen. **(C)** T2-weighted image shows a hyperintense mass pointed by the red arrowheads in the left lower abdomen. **(D)** PET-CT shows no neoplasm indications. CT, computer tomography; MRI, magnetic resonance imaging; PET, positron emission tomography.

**Figure 3:**
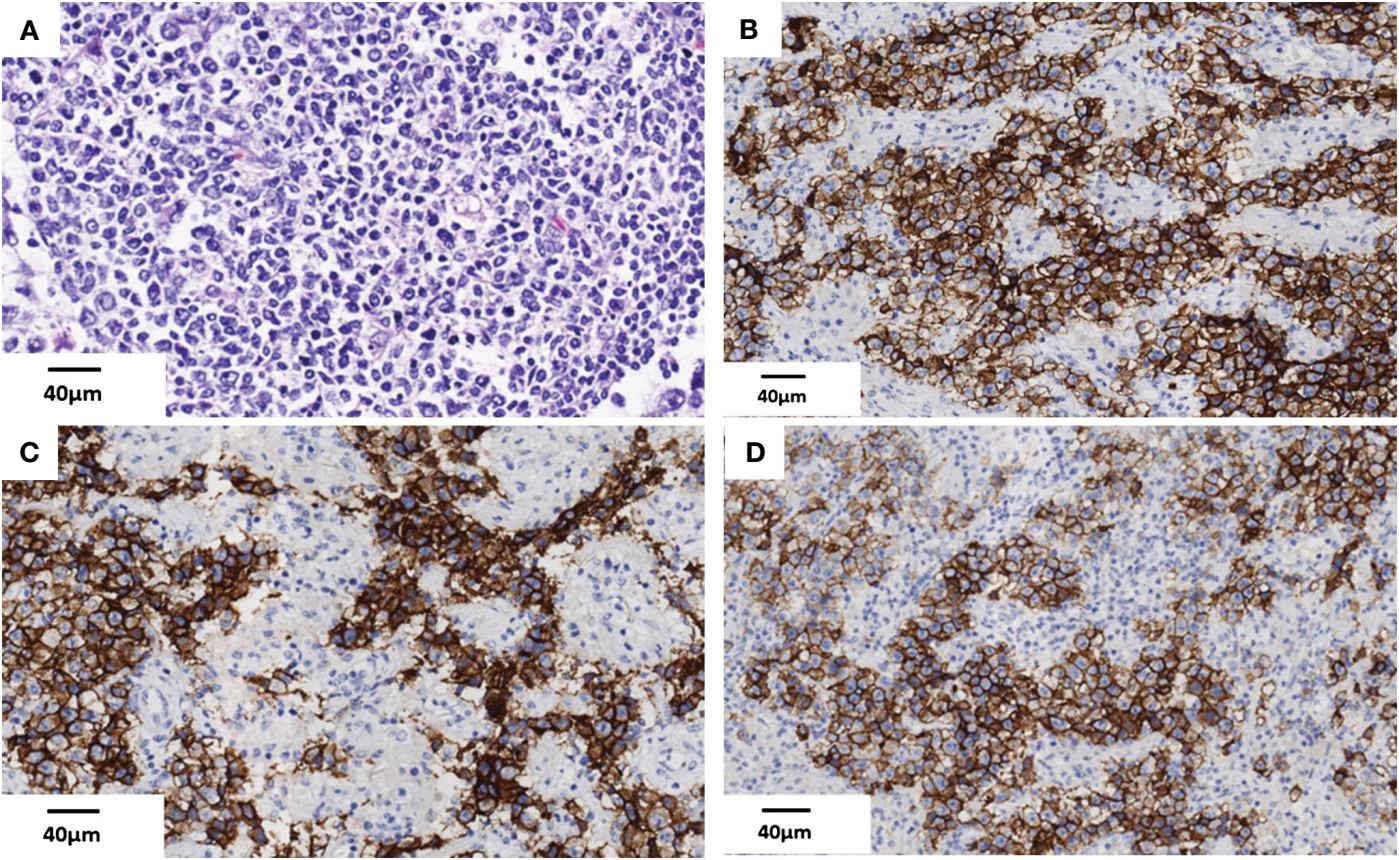
Histopathologic findings of the ovarian dysgerminoma in this case. **(A)** Tumor cell nests are located in the ovarian parenchyma (HE) (scale bar 40μm). **(B–D)** Immunohistochemistry of tumor tissues show CD117 (+), SALL4 (+), and OCT3/4 (+) in this case (scale bar 40μm). HE, hematoxylin and eosin.

The patient experienced discomfort in the lower abdomen while undergoing methylprednisolone treatment. Abdominal palpation revealed a round, hard mass in the left lower abdomen with poor mobility, but without any tenderness or rebound pain. Abdominal magnetic resonance imaging (MRI) and computed tomography (CT) showed a tumor in the left ovarian. An equal-density mass was seen on the abdominal CT (**Figure 2A**). Additionally, a T2 hyperintense (**Figure 2B**) and a T1 hypointense (**Figure 2C**) lesion in the left lower abdomen was discovered by abdominal MRI. Moreover, the cancer biomarkers [alpha-fetoprotein (AFP), carcinoembryonic antigen (CEA), carbohydrate antigen 125 (CA-125), and CA-199] were detected, but the tumor markers were negative. After undergoing a pathologic biopsy and surgical resection, the neoplasm was diagnosed as an ovarian dysgerminoma (**Figure 3A**). Immunohistochemistry of tumor tissues showed positive results for cluster of differentiation 117 (CD117), spalt-like transcription factor 4 (SALL4), and octamer-binding transcription factor 3/4 (OCT3/4), while negative for epithelial membrane antigen (EMA), CD3, CD20, CD30, pair box gene 8 (PAX-8), human melanoma black 45 (HMB-45), anaplastic lymphoma kinase (ALK), and placental alkaline phosphatase (PLAP) (**Figures 3B-D**). The ovarian mass was confirmed as a malignant tumor after the pathological biopsy. As a result, the patient had four rounds of chemotherapy and stopped using immunosuppressive medication.

The patient underwent a positron emission tomography (PET) scan one month after the final chemotherapy treatment. The PET-CT scan, used to assess the course of treatment after the most recent round of chemotherapy, showed no indications of the tumor (**Figure 2D**). Moreover, the patient’s vision returned to normal. We rechecked that AQP4-IgG in the serum of the patient was negative (**Figure 1C**). Throughout the following two years of follow-up, the patient remained free from any abnormal symptoms.

## 3 Diagnostic assessment

The initial clinical features were similar to the presentation of ophthalmic diseases. However, ophthalmic examinations, MRI results, and VEP findings revealed optic nerve damage without any lesions in the eyes. Therefore, we suspected that the patient had NMOSD. Subsequent testing found that the antibodies for NMOSD were positive in the serum, confirming the diagnosis of NMOSD. The patient responded well to immunosuppressive treatment. The combination of abdominal symptoms, physical signs, and findings from both CT and MRI scans strongly indicated the presence of a left ovarian tumor in the patient. Then the pathologic biopsy and immunohistochemistry of tumor tissues confirmed the diagnosis of ovarian dysgerminoma. Thus, the patient was diagnosed as paraneoplastic NMOSD overlapping with ovarian dysgerminoma.

## 4 Literature review

A literature search was performed using the PubMed and Web of Science database. The following combinations of search terms were used: “neuromyelitis optica spectrum disorder and ovarian neoplasms”, and “NMOSD and ovarian neoplasms”. The search was limited to articles in English. A review was done of the information that was available in full-text or abstract form, along with any relevant citations and references. We summarized the clinical manifestations of all nine cases with paraneoplastic NMOSD associated with ovarian neoplasms are presented in **Table 1**.

**Table 1:** Characteristics of patients with overlapping NMOSD and ovarian neoplasms.

Country	Publication date	Sex	Age (years)	Types of neoplasms	NMOSD phenotypes	MRI findings	Auto-antibodies	Tumor pathology	Duration of follow-up	Treatment of NMOSD	Treatment of neoplasms	Prognosis
Spina (7)	2013	F	43	Ovarian teratoma	APS, LETM	Demyelinating lesions in the brainstem	AQP4 (+)	AQP4, CD20, CD4, CD3, and CD138	1 year	IVMP + TPE + IVIg	Surgery	Good
Canada (8)	2018	F	54	Ovarian serous adenocarcinoma	APS	Demyelinating lesions from the medulla to spinal cord	AQP4 (+)	AQP4	2 years	IVMP + TPE + mitoxantrone	Surgery + chemotherapy	Good
Italy (9)	2013	F	50	Ovarian teratoma	LETM, BS	Demyelinating lesions in the pons, hypothalamus, medulla oblongata, and cervical spine	AQP4 (+)	NA	17 months	IVMP + TPE + AZA	Surgery	Good
France (10)	2019	F	15	Ovarian teratoma	APS, LETM	Demyelinating lesions in the brainstem, and a cervical spinal cord	AQP4 (+)	AQP4, CD3, CD20, and CD8	2 years	IVMP + Rituximab	Surgery	Good
		F	21	Ovarian teratoma	APS, LETM	Demyelinating lesions in the hypothalamus, temporal lobes, area postrema, and spinal cord	AQP4 (+)	AQP4, CD3, CD20, and CD8	9 months	IVMP + TPE + Rituximab	Surgery	Important visual sequelae
		F	41	Ovarian teratoma	APS, LETM	Demyelinating lesions in the spinal cord	AQP4 (+), GFAP (+)	AQP4, CD3, CD20, and CD8	2 years	IVMP + MMF	Surgery	Mild sensory abnormalities
India (11)	2019	F	46	Ovarian adenocarcinoma	ON, LETM	Demyelinating lesions in the cervical cord	AQP4 (+)	NA	6 months	IVMP + AZA	Chemotherapy + surgery	Limb weakness
Japan (12)	2021	F	27	Ovarian teratoma	APS, LETM, ON	Demyelinating lesions in spinal cord and pons	AQP4 (+)	AQP4, CD8, CD45, CD20, and CD138	16 months	IVMP+ tacrolimus	Surgery	Good
China (our case)	2024	F	48	Ovarian dysgerminoma	ON	Normal	AQP4 (+)	CD117, SALL4, and OCT3/4	2 years	IVMP + MMF	Surgery + chemotherapy	Good

APS, area postrema syndrome; AQP4, aquaporin 4; AZA, azathioprine; CD, cluster of differentiation; F, female; IVMP, intravenous methylprednisolone; GFAP, glial fibrillary acidic protein; IVIg, intravenous immunoglobulin; LETM, longitudinally extensive transverse myelitis; MMF, mycophenolate mofetil; NA, not available; NMOSD, neuromyelitis optica spectrum disorder; ON, optic neuritis; TPE, therapeutic plasma exchange; BS, brainstem; (+), positive.

## 5 Discussion

We described a unique clinical case of NMOSD associated with ovarian dysgerminoma for the first time. NMOSD is a rare auto-immune disease causing multifocal central nervous system (CNS) inflammation (13). AQP4-IgG was detected in more than 80% of patients with NMOSD (3). Approximately 5% of NMOSD with malignancies (such as breast and ovarian cancer) were considered as paraneoplastic NMOSD (14). However, the mechanisms of the paraneoplastic NMOSD are still unclear.

Intratumorally neural tissue that expresses AQP4 may be a mediating factor in paraneoplastic NMOSD linked to malignancy (15). Previous studies reported that a part of patients with ovarian teratoma expressed N-methyl-D-aspartate receptor (NMDAR) and AQP4-IgG (12, 16). Moreover, in addition to GFAPα-IgG, the coexistence of AQP4-IgG and/or anti-NMDAR was detected in most of the autoimmune GFAP astrocytopathy patients with teratomas (16). These investigations revealed a common pathogenesis between CNS autoimmune disorders and ovarian teratomas (12). In this case, the AQP4-IgG was detected positive in the serum using CBA method. Furthermore, both ovarian dysgerminoma and ovarian teratoma are malignant ovarian tumors derived from germ cell. Therefore, we hypothesize that ovarian dysgerminoma can develop into paraneoplastic NMOSD induced by malignancy-associated autoimmune mechanisms, similar ovarian teratoma. However, to our knowledge, up to now, there is no research on the relationship between ovarian dysgerminoma and neurologic demyelinating diseases. Thus, the assumption of AQP4 mediating ovarian dysgerminoma needs further study to identify.

The reports presented in **Table 1** summarize on all nine patients with NMOSD associated with ovarian neoplasms. In the presented case, all nine female patients with positive AQP4-IgG. The age at onset of this case of paraneoplastic NMOSD, typically around 50 years old, was consistent with the clinical characteristic of paraneoplastic NMOSD with ovarian neoplasms. Most ovarian tumors linked to paraneoplastic NMOSD were teratomas, with this study uniquely reporting a singular case of NMOSD in conjunction with an ovarian dysgerminoma. The patient only had the clinical symptoms of optic neuritis without myelitis or brainstem lesion, which is inconsistent with the clinical features of NMOSD overlapping with ovarian teratomas. Most case with paraneoplastic NMOSD associated with ovarian neoplasms revealed demyelinating lesions in the central nervous system. However, the MRI of this case did not detect any demyelinating lesions. The clinical manifestations of NMOSD overlapping with ovarian dysgerminoma requires more studies to summarize.

Overall, patients with paraneoplastic NMOSD associated with ovarian neoplasms had a favorable prognosis after immunosuppressive therapy. However, no unified guideline on the treatment of paraneoplastic NMOSD was reported in the literature. Intravenous methylprednisolone (IVMP) is the most commonly used treatment for paraneoplastic NMOSD and has demonstrated excellent therapeutic efficacy (5). Regarding primary tumor treatments, surgical resection and chemoradiotherapy may improve neurological symptoms (10, 12). In this case, the serum AQP4-IgG was negative after immunosuppressive therapy and tumor surgery. Previous studies have found that the clinical symptoms of patients with ovarian teratoma were significantly improved after resection, and the titer of AQP4 antibody in serum decreased or became negative (12). However, it is not clear whether this phenomenon is caused by immunosuppressive therapy or tumor removal. Moreover, it is unclear if patients with paraneoplastic NMOSD require ongoing immunosuppressive medication following surgery. On the one hand, immunosuppressive therapy is useful in avoiding NMOSD recurrence; on the other hand, several immunosuppressive drugs, such as azathioprine and mitoxantrone, have been linked to cancer recurrence and metastasis (8). In our study, the patient terminated immunosuppressive therapy prior to tumor surgery, and after a half-year follow-up, the tumor and NMOSD did not recur. The result suggests that ovarian dysgerminoma may trigger paraneoplastic NMOSD. When the primary tumor was removal, NMOSD caused by autoimmune abnormalities also improved.

Our case report has some limitations. First, the result of AQP4 expression in this case of the ovarian dysgerminoma was missed. Previous studies have uncovered AQP4 expression in ovarian neoplasm tissues (7, 8, 10, 12). This evidence indicates that ovarian neoplasms might mediate paraneoplastic NMOSD through AQP4 expression. Regrettably, we did not check for AQP4 expression in the tumor slices in our case, leaving the relationship between the ovarian dysgerminoma and paraneoplastic NMOSD unclear. Second, we did not find any abnormal MRI findings. Enhanced MRI of the optic nerve or optical coherence tomography (OCT) may have revealed optic nerve damage to support the diagnosis.

In summary, we reported a case with ovarian dysgerminoma overlapping NMOSD for the first time. It is crucial to detect ovarian tumor for patients with NMOSD. The clinical features of patients with ovarian dysgerminoma overlapping NMOSD requires more studies to summarize. Moreover, our study suggests that the treatment of primary tumors is crucial, and long-term immunosuppressive therapy may not be necessary for paraneoplastic NMOSD.

## Data availability statement

The original contributions presented in the study are included in the article/supplementary material. Further inquiries can be directed to the corresponding author/s.

## Ethics statement

The studies involving humans were approved by The Ethics Committee and the Expert Committee of The Central Hospital of Shaoyang. The studies were conducted in accordance with the local legislation and institutional requirements. The participants provided their written informed consent to participate in this study. Written informed consent was obtained from the individual(s) for the publication of any potentially identifiable images or data included in this article.

## Author contributions

PL: Data curation, Formal analysis, Writing – original draft, Writing – review & editing. SW: Data curation, Writing – review & editing. CZ: Formal analysis, Methodology, Writing – review & editing. YL: Conceptualization, Data curation, Methodology, Writing – review & editing.
